# International development of four EORTC disease-specific quality of life questionnaires for patients with Hodgkin lymphoma, high- and low-grade non-Hodgkin lymphoma and chronic lymphocytic leukaemia

**DOI:** 10.1007/s11136-017-1718-y

**Published:** 2017-11-10

**Authors:** Lonneke van de Poll-Franse, Simone Oerlemans, Anne Bredart, Charalampia Kyriakou, Monika Sztankay, Stephan Pallua, Laurien Daniëls, Carien L. Creutzberg, Kim Cocks, Sandra Malak, Giovanni Caocci, Stefano Molica, Weichu Chie, Fabio Efficace, on behalf of the EORTC Quality of Life Group

**Affiliations:** 1 Department of Research, Netherlands Comprehensive Cancer Organisation (IKNL), Utrecht, The Netherlands; 2 Department of Psychosocial Research, Division of Psychosocial Research & Epidemiology, The Netherlands Cancer Institute, Amsterdam, The Netherlands; 3 Department of Medical and Clinical Psychology, Tilburg University, Tilburg, The Netherlands; 4 Psycho-Oncology Unit, Institut Curie, Paris, France; 5 Psycho-pathology and Health Process Laboratory Psychology Institute, University Paris Descartes, Paris, France; 6 Royal Free and North West London Hospitals, National Health Service Trust, London, UK; 7 Department of Psychiatry, Psychotherapy and Psychosomatics, Innsbruck Medical University, Innsbruck, Austria; 8 Department of Radiation Oncology, Leiden University Medical Centre (LUMC), Leiden, the Netherlands; 9 KCStats Consultancy, York, UK; 10 University of York, York, UK; 11 Hôpital René Huguenin-Institut Curie- Hématologie, Saint-Cloud, France; 12 Hematology, Department of Medical Sciences, University of Cagliari, Cagliari, Italy; 13 Azienda Ospedalier Ciaccio, Catanzaro, Italy; 14 National Taiwan University, Taipei, Taiwan; 15 Health Outcomes Research Unit, Italian Group for Adult Hematologic Diseases (GIMEMA) Data Centre, Rome, Italy

**Keywords:** Quality of life, Symptoms, Hodgkin lymphoma, Non-Hodgkin lymphoma, Chronic lymphocytic leukaemia

## Abstract

**Purpose:**

This paper describes the international, cross-cultural development of four disease-specific EORTC QoL questionnaires, to supplement the EORTC QLQ-C30, for patients with Hodgkin lymphoma (HL), high- or low-grade non-Hodgkin lymphoma (HG/LG-NHL), and CLL.

**Methods:**

Questionnaire development was conducted according to guidelines from the EORTC Quality of Life Group. Phase I comprised generation of QoL issues relevant to patients. Phase II included operationalization and assessment of item relevance. In phase III, items were pretested in a cross-cultural sample.

**Results:**

In Phase I, 75 issues were identified through focus groups and systematic literature searches. Interviews with 80 health-care professionals and 245 patients resulted in a provisional module of 38 items (phase II) representing items relevant for all or at least one of the four malignancies. In Phase III, this was tested in 337 patients from five European countries and resulted in a questionnaire with 27 items for HL (EORTC QLQ-HL27), 29 items for HG-NHL (EORTC QLQ-NHL-HG29), 20 items for LG-NHL (EORTC QLQ-NHL-LG20) and 17 items for CLL (EORTC QLQ-CLL17).

**Conclusions:**

This study provides four new EORTC modules for use in clinical research and routine practice in conjunction with the EORTC QLQ-C30 for assessing QoL in patients with lymphoma and CLL.

## Introduction

Treatment of patients with lymphoproliferative disorders, i.e. Hodgkin lymphoma (HL), high-grade (HG: aggressive) and low-grade (LG: indolent) non-Hodgkin lymphoma (NHL) and chronic lymphocytic leukaemia (CLL), has witnessed dramatic changes in the last two decades, leading to more prolonged, intensive treatments with improved survival rates and/or remission duration [1–4]. To date, more than 80% of patients diagnosed with HL are expected to be disease free at 5 years or more after diagnosis [4–6]. The overall 5-year relative survival rate for patients with NHL (2006–2012) is 63–82%,[1, 4, 6] depending on the NHL type, stage of disease at diagnosis, treatment and age of the patient.

Recently, a number of targeted therapies have been made available for NHL and CLL patients. For the latter group of patients, for example, these include monoclonal antibodies that have shown to be effective or are currently investigated in combination with other drugs. Results from the Swedish population-based Lymphoma Registry Study showed a significant survival improvement in patients diagnosed with Follicular Lymphoma between 2000 and 2010. This improvement correlated with the increasing use of first-line rituximab over time and with regional differences in first-line rituximab use [2]. The Swedish observation is in line with dramatic improvements of relative survival in the last decade for NHL patients as recently reported from the SEER database [4].

Despite the changing landscape of treatment of lymphoproliferative disorders and in contrast to the large number of quality of life (QoL) studies in patients with solid tumours, relatively few studies have reported QoL in patients with haematological malignancies [7]. Also, the American Society of Hematology (ASH) has voiced concern about the lack of data in this area, advocating urgent efforts to raise the standards of QoL research [8]. Likewise, international recommendations for various hematologic diseases are also now paying greater attention to QoL assessment [8–12]. Studies investigating effects and complications after haematological cancer treatment have identified problems in several domains, including eye, oral, endocrine, neurosensory and cardiopulmonary impairments [13–20]. The FACT-Lymphoma questionnaire has been developed more than a decade ago to assess QoL in the broad group of all subtypes of lymphoma cancer patients. From a pool of 69 items, following expert relevancy ratings (*n* = 17), patient input (*n* = 75) and item correlations, a lymphoma subscale of 15 items was constructed [21]. This questionnaire covers some, but not all issues that are sometimes only relevant to subtypes of lymphoma patients (e.g. HL, HG or LG-NHL).

A number of trials conducted in patients with hematologic malignancies have typically used cancer-specific or general measures such as the EORTC QLQ-C30 [22] or the SF-36 [23–25]. This possibly limits a full appraisal of outcome differences between arms. It is envisaged that use of haematological cancer-specific questionnaires would increase sensitivity to detect functional limitations and symptoms in future trials of patients with lymphoproliferative disorders. While new emerging therapies for these patients are enlarging therapeutic options, we need standardized and validated tools to measure the impact of these on QoL and symptom burden.

The main objective of this international study was to develop questionnaires (to be used in conjunction with the EORTC QLQ-C30) to more comprehensively assess QoL of patients with HL, HG/LG-NHL or CLL. As differences in age at disease onset, treatment and survival outcomes among these cancers may influence QoL, a secondary objective was to evaluate whether it would be possible to develop only one questionnaire, covering all relevant issues for these patients, or whether their QoL issues are sufficiently different to warrant the development of separate questionnaires.

## Methods

### Study design and patients

**Fig. 1:**
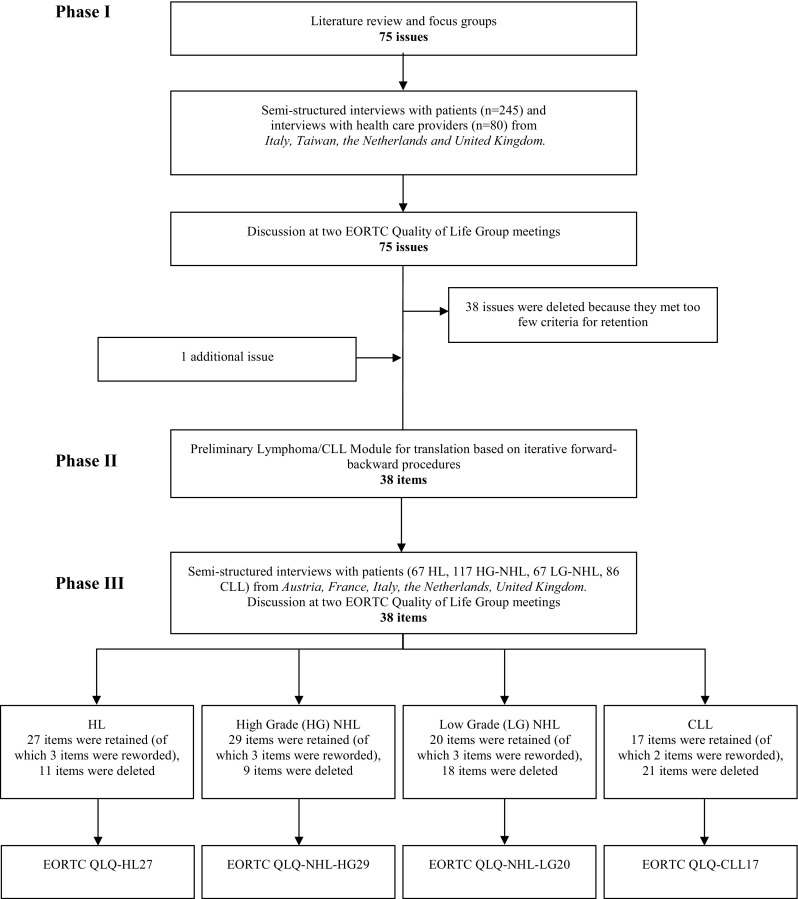
Summary of module development

Questionnaire development was conducted according to guidelines from the EORTC Quality of Life Group [26]. These guidelines consist of four phases: (1) generation of QoL issues through literature searches, focus groups and interviews with patients and health-care professionals (HCPs); (2) operationalization and assessment of item relevance, issues derived from phase 1 were operationalized into items according to the response format and time frame of the EORTC QLQ-C30; (3) pretesting the questionnaire module among a new and larger sample of lymphoma and CLL patients from five countries to identify problems regarding wording and comprehensiveness; (4) large-scale international field testing. This paper presents the phase 1–3 results and phase 4 will be carried out in a future study. The main steps of the whole development process are summarized in Fig. 1.

Eligibility criteria were adult patients (> 18 years) with a diagnosis of HL, HG/LG-NHL or CLL either with current or past treatment for primary or relapsed disease, who were able to understand and speak the local language. Patients with psychiatric disorders or major cognitive dysfunctions were excluded. Ethical approval from each participating centre was obtained and all patients provided written informed consent.

#### Phase 1

Extensive literature searches through PUBMED and PsychINFO were conducted to identify all relevant HRQoL issues in the last 15 years, to include issues of patients treated longer ago but also to include symptoms as a result of newer therapies. In addition, two focus groups among Dutch CLL/NHL (*N* = 9) and HL (*N* = 7) patients were composed in order to discuss relevant HRQoL issues in general, with respect to their phase of disease. We also added issues that were included in the earlier developed EORTC QLQ-CLL16, but that were not yet in our list.

The list of issues that was gathered was evaluated in semi-structured interviews with patients and health-care professionals (HCPs). Patients and HCPs ratings of each item were collected for the following criteria (1) relevance, which was rated on a four-point likert scale ranging from ‘not at all relevant’ (1 point) to ‘very much relevant’ (4 points) and it refers to the frequency with which a problem or symptom occurs and the trouble it may cause. (2) priority for inclusion (yes/no), which was rated for each item to identify those items that affect patients’ HRQoL most and should definitely be included in the final questionnaire. (3) breadth of coverage was investigated by asking patients and HCPs to suggest any relevant issues, which were not included in the item list and should therefore be added.

#### Phase 2

In Phase 2, the issues derived from Phase 1 were operationalized into items according to the response format and time frame of the EORTC QLQ-C30. Rather than developing new items, if available, items that matched content in the modules were selected from the EORTC Quality of Life Group Item Library.

#### Phase 3

The pretesting of the module was tested among a new and larger sample of lymphoma and CLL patients from five countries to identify problems regarding wording and comprehensiveness. In this Phase, first patients completed the general EORTC QLQ-C30 and the provisional lymphoma/CLL-specific module. Then they took part in a semi-structured interview where they were asked if any items were annoying, confusing, upsetting or intrusive, and if there were irrelevant or missing issues.

### Data analyses and criteria for item selection

Results from phase 1 and 3 interviews were analysed using descriptive statistics according to the EORTC guidelines and data were analysed per tumour group. Analyses were performed with IBM SPSS statistics version 19.

Phase 1 issues were retained if patients reported a mean score of ≥ 1.75 (range 1–4) for the relevance question and > 15% of patients considered the item a priority, and HCPs reported a mean score of ≥ 2.00 for the relevance question and > 25% considered it a priority. For patients, two points were assigned for every criterion that was met and for HCPs, one point was assigned. This was done to give more importance to the results of patients. Subsequently, issues that had a score of 4, 5 or 6 points for at least one of the four tumour groups were selected for inclusion in the provisional phase 3 item questionnaire.

In phase 3, the following criteria were used for item selection: (1) patients reported a mean score ≥ 1.6 (range 1–4); (2) prevalence ratio (number of patients scoring 2: ‘a little’, 3: ‘quite a bit’ or 4: ‘very much’ divided by the total number who completed the item) ≥ 30%; (3) range of scores > 2 points; (4) responses in categories 3 and 4 ≥ 15%; (5) at least 95% response to item; (6) no more than 3% of patients expressed significant concerns of a particular item (e.g. item is upsetting, confusing); (7) consistency across languages and cultures (mean and prevalence). Items were retained in the list if (1) four or five criteria were met from the first five criteria; (2) no concerns were expressed by > 3% of patients on an item; (3) consistency across countries was observed [26]. A hypothesized scale structure was developed based on content. Descriptive statistics and preliminary psychometric testing of the hypothesized scales included evaluation of internal consistency (Cronbach’s α) and clinical validity (known-group comparisons). We hypothesized that patients on treatment would report worse symptom burden, fatigue, physical function, emotional impact and worries compared to patients off treatment. Based on previous findings, we furthermore hypothesized that older patients would report lower scores on emotional impact and worries compared to younger patients. Full psychometric testing requires larger patient numbers and will be performed in phase 4 of the module development.

## Results

### Phase 1: generation of issues

Extensive literature searches through PUBMED and PsychINFO (January 2011) resulted in thirteen studies [13–20, 27–31] performed between 1998 and 2011 that formed the basis of the provisional list as no new issues came out of further searches of other databases. Also, previous work that had been conducted by EORTC QoL group members on the development of the EORTC QLQ-CLL16 was included and updated in the module development process. Focus group interviews were held and any issues identified through the focus groups but not came out of literature search were added to the provisional list. This extensive process yielded an initial list of 75 potential relevant issues.

This list with 75 issues was used as a basis to conduct semi-structured interviews with 245 patients, of whom 75 had a diagnosis of HL, 66 of high-grade (aggressive) non-Hodgkin lymphoma (HG-NHL), 41 of low-grade (indolent) non-Hodgkin lymphoma (LG-NHL) and 63 had a diagnosis of Chronic Lymphocytic Leukaemia (CLL). Patients were recruited in four different countries; 100 from Italy, 48 from the UK, 83 from the Netherlands, and 14 from Taiwan. Sociodemographic and clinical characteristics of patients participating in phase 1 are shown in Table 1.

**Table 1:** Sociodemographic and clinical characteristics of *phase I* participating patients with HL, HG-NHL, LG-NHL or CLL

	HL (*N* = 75)	HG-NHL (*N* = 66)	LG-NHL (*N* = 41)	CLL (*N* = 63)
Country (language)				
Italy (Italian)	26 (35)	24 (36)	17 (41)	33 (52)
Netherlands (Dutch)	30 (40)	16 (24)	14 (34)	23 (37)
Taiwan (Taiwanese)	1 (1)	9 (14)	3 (7)	1 (1)
UK (English)	18 (24)	17 (26)	7 (17)	6 (10)
Age (years)				
Mean (SD)	40 (17)	55 (17)	62 (12)	68 (9)
Range	18–78	18–86	41–82	49–85
Sex				
Male	41 (55)	37 (57)	19 (48)	41 (66)
Female	33 (45)	28 (43)	21 (53)	21 (34)
Time since diagnosis (years)				
Mean (SD)	3.4 (3.6)	2.3 (3.1)	5.2 (4.3)	4.1 (3.9)
< 2 years	30 (41)	40 (61)	12 (30)	14 (23)
2–5 years	25 (34)	13 (21)	6 (15)	28 (46)
> 5 years	19 (26)	13 (20)	23 (56)	19 (31)
Range	0–13	0–12	0–15	0–18
Treatment received				
Radiotherapy	26 (35)	16 (24)	10 (24)	0 (0)
Chemotherapy	73 (97)	64 (97)	33 (81)	33 (52)
Stem cell transplantation	6 (8)	3 (5)	2 (5)	0 (0)
Monoclonal anti bodies	0 (0)	41 (62)	21 (51)	12 (19)
Active surveillance	0 (0)	1 (2)	6 (15)	34 (54)
On treatment at time of issue list				
Yes	10 (14)	23 (35)	16 (39)	17 (27)
Stage				
Ann Arbour/RAI	Ann Arbour	Ann Arbour	Ann Arbour	RAI
I/0	5 (7)	16 (24)	2 (5)	14 (22)
II/1	33 (44)	8 (12)	6 (15)	4 (6)
III/2	21 (28)	6 (9)	6 (15)	6 (10)
IV/3	6 (8)	21 (32)	17 (42)	4 (6)
/4	–	–	–	2 (3)
Unknown	10 (13)	15 (23)	10 (24)	33 (52)

The list of 75 issues was also used as a basis to conduct semi-structured interviews with HCPs (haematologists, radiation oncologists, medical oncologists, nurses and psychologists), all of whom were experienced in working with Hl, NHL or CLL patients. Thirty HCPs completed the list for HL, 29 HCPs for NHL and 21 HCPs completed the list for CLL. HCPs were recruited from the same countries as patients.

Of the 75 issues, 36 met all inclusion criteria relating to relevance, priority and breadth of coverage for at least one of the four tumour groups and these items were included in the phase III questionnaire. Thirty-seven issues did not meet the inclusion criteria in any of the tumour groups and were deleted. Furthermore, one issue was added about ‘worries on treatment causing future health problems’ to assess health problems in general as patients reported many symptoms.

### Phase 2: operationalization of the provisional questionnaire

The 37 issues from phase 1 were formulated into 38 questions (i.e. items) using the EORTC standard formatting. The issue ‘hair loss’ was split up in two questions. For 20 items, formulation was already available within the EORTC item library. The Phase 1 and 2 development process report was peer reviewed and formally approved by the EORTC QLG module development committee (MDC) before starting with phase 3.

### Phase 3: pretesting of the provisional questionnaire for relevance and acceptability

The provisional lymphoma/CLL module containing 38 items and the EORTC QLQ-C30 were completed by 67 patients with HL, 117 patients with HG-NH, 67 patients with LG-NHL and 86 patients with CLL, and debriefing interviews were held with all patients. Included patients were different from those who participated in phase 1. Patients were recruited in five countries, i.e. 88 from Italy, 78 from the UK, 81 from the Netherlands, 76 from France and 14 from Austria. Sociodemographic and clinical characteristics of patients included in phase 3 are shown in Table 2.

**Table 2:** Sociodemographic and clinical characteristics of *phase III* participating patients with HL, HG-NHL, LG-NHL or CLL

	HL (*N* = 67)	HG-NHL (*N* = 117)	LG-NHL (*N* = 67)	CLL (*N* = 86)
Country (language)				
Austria (German)	2 (3)	10 (9)	1 (2)	1 (1)
France (French)	10 (15)	32 (27)	19 (28)	15 (17)
Italy (Italian)	16 (24)	28 (24)	12 (18)	32 (37)
Netherlands (Dutch)	21 (31)	26 (22)	16 (24)	18 (21)
UK (English)	18 (27)	21 (18)	19 (28)	20 (23)
Age (years)				
Mean (SD)	44 (16)	59 (17)	65 (11)	69 (10)
Range	18–79	23–89	34–87	35–87
Sex				
Male	32 (48)	68 (58)	36 (54)	59 (69)
Female	35 (52)	49 (42)	31 (46)	27 (31)
Time since diagnosis (years)				
Mean (SD)	5.6 (7.1)	2.8 (3.2)	3.9 (4.1)	5.0 (4.4)
< 2 year	20 (30)	52 (44)	24 (36)	20 (23)
2–5 years	24 (36)	45 (39)	27 (40)	30 (35)
> 5 years	22 (33)	19 (16)	16 (24)	35 (41)
Treatment received				
Radiotherapy	31 (46)	19 (16)	5 (8)	0 (0)
Chemotherapy	66 (99)	113 (97)	61 (91)	51 (59)
Stem cell transplantation	11 (16)	20 (17)	5 (8)	1 (1)
Monoclonal antibodies	3 (5)	54 (47)	33 (49)	24 (28)
Active surveillance	6 (9)	17 (15)	15 (22)	38 (44)
No. of treatment lines				
One line	34 (51)	70 (60)	38 (57)	62 (72)
Two or more lines	33 (49)	46 (40)	29 (43)	24 (28)
On treatment at time of issue list				
Yes	26 (39)	56 (48)	45 (67)	45 (52)
No	41 (61)	60 (52)	21 (31)	31 (36)
Stage of disease				
Ann Arbour/RAI	Ann Arbour	Ann Arbour	Ann Arbour	RAI
I/0	8 (12)	7 (6)	5 (8)	24 (28)
II/1	23 (34)	10 (9)	3 (5)	8 (9)
III/2	14 (21)	24 (21)	12 (18)	14 (16)
IV/3	13 (19)	44 (38)	35 (52)	13 (15)
4	–	–	–	6 (7)
Unknown/not determined	9 (13)	32 (28)	12 (18)	21 (24)
Comorbidity				
Yes	35 (52)	71 (61)	50 (75)	63 (73)
Karnofsky score				
0–40%	0 (0)	1 (1)	0 (0)	0 (0)
50–70%	7 (10)	20 (17)	6 (9)	8 (9)
80–100%	56 (84)	81 (69)	58 (87)	74 (86)
Missing	4 (6)	15 (13)	3 (5)	4 (5)
Living arrangement				
Living with partner/family	45 (75)	77 (68)	46 (69)	61 (71)
Living with others	11 (18)	26 (23)	12 (18)	12 (14)
Living alone	2 (3)	11 (10)	7 (10)	11 (13)
Education				
No or primary school	3 (5)	11 (10)	10 (15)	20 (23)
Secondary education	27 (40)	49 (43)	31 (46)	34 (40)
Pre-university training	31 (51)	54 (47)	23 (34)	29 (34)
Employment				
Yes	28 (45)	36 (31)	18 (29)	15 (17)
No (incl. retired, homemaker)	34 (55)	76 (69)	45 (71)	66 (83)

Since large differences were observed in the retention of items for the four tumour groups as shown in Table 3, results are presented per disease type. Some items were not relevant or even upsetting to certain subgroups, while very relevant to others. In order to create relevant and at the same time not upsetting questionnaires, we decided to continue the development of four disease-specific questionnaires.

**Table 3:** Items included in the EORTC QLQ-HL27, EORTC QLQ-NHL-HG29, EORTC QLQ-NHL-LG20 and EORTC QLQ-CLL17. Item number starts from #31 as the EORTC QLQ-C30 (which should be used in conjunction with these questionnaires) has 30 items

	EORTC QLQ-HL27	EORTC QLQ-NHL-HG29	EORTC QLQ-NHL-LG20	EORTC QLQ-CLL17
Each item is rated on a four-point scale: not at all, a little, quite a bit and very much				
31. Have you had muscle weakness?	**■**	**■**	**■**	**■**
32. Have you had aches or pains in your muscles or joints?	**■**	**■**	**■**	**■**
33. Have you had aches or pain in your bones?	**■**	**■**		**■**
34. Have you had a dry cough?		**■**		
35. Have you had a dry mouth?		**■**	**■**	**■**
36. Have you had problems with your sense of taste?	**■**	**■**	**■**	
37. Have you lost any hair?				
38. Answer this question only if you lost any hair: have you been upset by the loss of your hair?				
39. Have you had vulnerable veins (for example, when having blood taken or receiving treatment)?	**■**			
40. Have you felt ill or unwell?		**■**		**■**
41. Have you had itching of your skin?	**■**			
42. Have you had night sweats?				**■**
43. Have you lost weight?				
44. Have you had tingling hands or feet?		**■**		
45. Have you had numbness in your fingers or toes?		**■**		
46. Have you had shortness of breath on exertion?	**■**	**■**	**■**	**■**
47. Have you felt you had setbacks in your physical condition?		**■**		
48. Have you had a lack of energy?	**■**	**■**	**■**	**■**
49. Have you felt drowsy?	**■**	**■**	**■**	**■**
50. Have you had sudden tiredness?	**■**	**■**	**■**	**■**
51. Have you had mood changes?	**■**	**■**		
52. Have you felt a lack of confidence in your body?	**■**	**■**	**■**	
53. Have you felt restless or agitated?	**■**		**■**	
54. Have you been dissatisfied with how your body functions?	**■**	**■**	**■**	
55. Have you lacked self-confidence?	**■**			
56. Have you had difficulty accepting limitations due to the disease?	**■**	**■**	**■**	
57. Have you worried about dying?				
58. Have you worried about picking up an infection?	**■**	**■**	**■**	
59. Have you worried about your health in the future?	**■**	**■**	**■**	**■**
60. Have you worried about recurrence of your disease?	**■**	**■**	**■**	**■**
61. Have you worried about becoming chronically ill?	**■**	**■**		
62. Have you worried about becoming dependent on others?	**■**	**■**	**■**	**■**
63. Have you worried about getting another type of cancer?	**■**	**■**	**■**	**■**
64. Have you worried about your treatment causing future health problems?	**■**	**■**	**■**	**■**
65. Have you worried about damage to your heart and blood vessels?	**■**	**■**		
66. If applicable: Have you had problems at your work or place of study due to the disease?	**■**	**■**	**■**	**■**
67. If applicable: Have you worried about not being able to continue working or your education?	**■**	**■**	**■**	**■**
68. If applicable: Have you been concerned about your ability to have children?	**■**	**■**		
Total items to include	**27**	**29**	**20**	**17**

#### Hodgkin lymphoma

Of the 38 items in the provisional module, 27 items met all inclusion criteria and were retained in the final module. Eleven items were deleted, because ten items (i.e. 34, 35, 37, 38, 40, 42–45, 47) did not meet all inclusion criteria and item 57 was rated as too upsetting by 4.5% of patients. Four items (39, 46, 54, 59) needed small English formatting changes and were reworded. This resulted in a list of 27 items for phase IV interviews among patients with HL (EORTC QLQ-HL27; Table 3).

#### High-grade (HG: aggressive) non-Hodgkin lymphoma

Of the 38 items, 29 met all inclusion criteria and were retained in the final module. Nine items were deleted, because 8 items (i.e. 37–39, 41–43, 53, 55) did not meet all inclusion criteria and item 57 was rated as too upsetting by 3.4% of patients. Three items (46, 54, 59) needed small English formatting changes and were reworded. This resulted in a list of 29 items for phase IV interviews among patients with high-grade NHL (EORTC QLQ-NHL-HG29; Table 3).

#### Low-grade (LG: indolent) non-Hodgkin lymphoma

Of the 38 items, 20 met all inclusion criteria and were retained in the module. Eighteen items were deleted, because 16 items (i.e. 33, 34, 37–45, 47, 51, 55, 65, 68) did not meet all inclusion criteria, item 57 was rated as too upsetting by 1.5% of patients and 3% reported additional comments. Item 61 was deleted since low-grade NHL is already a chronic disease and this question is therefore not applicable to these patients. Furthermore, 4.5% of patients reported a comment about the word ‘chronic’ in this question. Three items (46, 54, 59) needed small English formatting changes and were reworded. This resulted in a list of 20 items for phase IV interviews among patients with low-grade NHL (EORTC QLQ-NHL-LG20; Table 3).

#### Chronic lymphocytic leukaemia

Of the 38 items, 17 met all inclusion criteria and were retained in the final module. Twenty-one items were deleted, because 19 items (i.e. 34, 36–39, 41, 43–45, 47, 51–56, 58, 65, 68) did not meet all inclusion criteria, item 57 was rated as too upsetting by 4.7% of patients and item 61 was deleted since CLL is already a chronic disease and 8% of patients reported a comment about the word ‘chronic’ in this question. Two items (46, 59) were reworded. This resulted in a list of 17 items for phase IV interviews among patients with CLL (EORTC QLQ-CLL17; Table 3).

### Proposed scale structure

Based on content and clinical relevance, three to five multi-item scales were proposed for the different questionnaires: Symptom burden due to disease and/or treatment, Neuropathy (as a distinct scale only in NHL-HG29), Physical condition/Fatigue, Emotional impacts (not in CLL17) and Worries/fears about health and functioning. Internal consistency ranged from 0.67 (only the symptom burden scale for NHL-LG20 was below 0.70) to 0.92 for the proposed scales indicating moderate to good internal consistency (Table 4).

**Table 4:** Proposed scale structure for the EORTC QLQ-HL27, EORTC QLQ-NHL-HG29, EORTC QLQ-NHL-LG20 and EORTC QLQ-CLL17 after phase III

Scale	Item	Cronbach’s α	Total sample	On treatment	Off treatment	≤ median age	> median age
			Mean (SD)	Mean (SD)	Mean (SD)	Mean (SD)	Mean (SD)
EORTC QLQ-HL27			*N *= 67	*N* = 26	*N *= 41	*N* = 34 (median age = 47)	*N *= 33 (median age = 47)
Symptom burden due to disease and/or treatment	Six items: 31–33, 36, 39, 41	0.71	22.2 (19)	26.2 (19)	19.7 (19)	18.2 (18)	26.3 (19)
Physical condition/fatigue	Four items: 46, 48–50	0.84	27.5 (25)	27.3 (25)	27.6 (26)	23.2 (24)	31.7 (26)
Emotional impacts	Six items: 51–56	0.89	22.6 (23)	25.2 (19)	21.0 (26)	23.7 (26)	21.5 (20)
Worries/fears health and functioning	11 items: 58–68	0.91/0.92^a^	29.8 (26)/ 31.2 (27)^a^	34.1 (26)/ 33.6 (24)^a^	29.3 (28)/ 27.4 (27)^a^	34.2 (29)/ 34.1 (27)	28.1 (26)/ 25.4 (24)

Scale	Item	Cronbach’sα	Total sample	On treatment	Off treatment	≤ median age	> median age
			Mean (SD)	Mean (SD)	Mean (SD)	Mean (SD)	Mean (SD)
EORTC QLQ-NHL-HG29			*N *= 117	*N *= 56	*N* = 60	*N* = 61 (median age = 61)	*N* = 56 (median age = 61)
Symptom burden due to disease and/or treatment	Seven items: 31–36, 40	0.77	27.3 (20)	33.2 (20)	22.2 (19)	30.1 (21)	24.4 (18)
Neuropathy	Two items: 44, 45	0.88	21.9 (28)	26.7 (28)	17.3 (28)	23.2 (29)	20.4 (27)
Physical condition/fatigue	Five items: 46–50	0.86	30.6 (25)	35.9 (20)	25.9 (23)	35.8 (25)	24.7 (23)
Emotional impacts	Four items: 51, 52, 54, 56	0.87	23.7 (25)	28.0 (27)	20.0 (22)	30.2 (26)	16.7 (22)
Worries/fears health and functioning	11 items: 58–68	0.90/0.90^a^	31.6 (25)/ 29.8 (24)^a^	35.5 (25)/ 33.5 (24)^a^	27.8 (25)/ 26.3 (25)^a^	40.2 (25)/ 39.4 (24)	22.2 (21)/ 19.3 (19)

Scale	Item	Cronbach’s α	Total sample	On treatment	Off treatment	≤ median age	> median age
			Mean (SD)	Mean (SD)	Mean (SD)	Mean (SD)	Mean (SD)
EORTC QLQ-NHL-LG20			*N* = 67	*N* = 45	*N* = 21	*N* = 38 (median age = 65)	*N *= 29 (median age = 65)
Symptom burden due to disease and/or treatment	Four items: 31, 32, 35, 36	0.67	24.1 (23)	27.8 (23)	16.8 (21)	21.1 (23)	28.0 (22)
Physical condition/Fatigue	Four items: 46, 48–50	0.80	25.0 (23)	29.3 (22)	16.7 (24)	23.9 (24)	26.4 (23)
Emotional impacts	Four items: 52–54, 56	0.87	22.5 (25)	26.6 (25)	14.7 (22)	22.1 (25)	22.9 (25)
Worries/fears health and functioning	Eight items: 58–60, 62–64, 66, 67	0.87/0.89^a^	30.1 (26)/ 29.7 (25)^a^	34.6 (27)/ 34.4 (27)^a^	22.0 (18)/ 21.0 (19)^a^	29.9 (25)/ 30.0 (24)	30.4 (27)/ 29.3 (27)

Scale	Item	Cronbach’s α	Total sample	On treatment	Off treatment	≤ median age	> median age
			Mean (SD)	Mean (SD)	Mean (SD)	Mean (SD)	Mean (SD)
EORTC QLQ-CLL17			*N* = 86	*N* = 45	*N *= 31	*N* = 47 (median age = 70)	*N* = 39 (median age = 70)
Symptom burden due to disease and/or treatment	Six items: 31–33, 35, 40, 42	0.85	25.2 (25)	25.4 (24)	23.7 (27)	29.5 (25)	20.0 (23)
Physical condition/Fatigue	Four items: 46, 48–50	0.87	25.4 (26)	28.8 (26)	21.0 (26)	28.6 (29)	21.6 (20)
Worries/fears health and functioning	Seven items: 59, 60, 62–64, 66, 67	0.88/0.86^a^	26.9 (25)/ 25.2 (25)^a^	33.9 (28)/ 32.3 (28)	18.7 (18)/ 16.8 (17)	33.9 (28)/ 32.3 (28)	18.7 (18)/ 16.8 (17)

^a^Cronbach’s α, mean and SD including the ‘if applicable’ questions (i.e. question 66, 67 and 68)

Comparisons of the mean scale scores between patients on and off treatment showed differences in all scales in the expected direction, with the strongest impact of treatment on symptom burden, neuropathy and physical condition among patients with LG/HG-NHL. Comparison between older and younger patients (dichotomized according to median age per subgroup) showed less emotional impact and worries about cancer and its treatment among the elderly in all subgroups. Symptom burden, physical condition and fatigue were more negatively impacted among the older HL and LG-NHL. In contrast, older HG-NHL and CLL patients experienced fewer symptoms and less impact on physical functioning than younger patients.

The phase 3 development process report was peer-reviewed and formally approved by the EORTC QLG module development committee (MDC), therefore the development can be taken forward to phase 4.

## Discussion

Four EORTC questionnaires to more comprehensively assess QoL in patients with HL (EORTC QLQ-HL27), NHL-HG (QLQ-NHL-HG29), NHL-LG (QLQ-NHL-LG20) and CLL (QLQ-CLL17) have been developed on an international basis. Large differences were observed in the mean and prevalence of items for the four tumour groups, where some items were relevant to certain subgroups, while at the same time upsetting to other subgroups. We therefore decided that four distinct questionnaires would result in better content validity and usability for future studies. After completion of phase 4 full psychometric testing, these questionnaires are to be used in conjunction with the EORTC QLQ-C30 core questionnaire and are expected to raise standards of outcome measurements in patients with lymphoproliferative disorders in future trials.

The EORTC approach that includes extensive literature review, and interviews with both patients and health-care providers from several different countries ensures content validity with cross-cultural relevance. This is particularly important for use in multicenter international studies.

Preliminary psychometric testing confirms the hypothesized multi-item scales, although full psychometric evaluation will be done in phase 4. Internal consistency was good (> 0.70) for all scales of all questionnaires, except the symptom burden scale of the NHL-LG20 questionnaire of showing a moderate internal consistency (0.67). Known-group comparisons of scores between patients on and off treatment and younger and older patients confirmed clinically relevant differences in outcomes. Our observation that older participants reported less emotional impact and fewer worries or fears about their health has been reported previously [32].

Importantly, full psychometric testing requires larger patient numbers and will be performed in a forthcoming prospective international field testing. Nevertheless, the phase 3 modules already receive a lot of attention as in recent years the treatment of patients with lymphoproliferative disorders has changed dramatically while at the same time international recommendations are advocating more research into the QoL of these patients [8–12]. The only other lymphoma questionnaire that currently exists is the Fact-Lym, that includes 15 lymphoma-specific items [21]. However, certain issues that we identified as relevant (e.g. neuropathic symptoms) are not included in the Fact-Lym that was developed in 2005.

In the past, treatment options for patients with lymphoma were limited to radiation and cytotoxic chemotherapies. In recent years, significant advances have been achieved in the understanding of the pathogenesis of lymphomas, leading to the emergence of a large number of new therapeutic agents targeting the signalling pathways, surface antigens of microenvironment. Rituximab was the first monoclonal antibody approved by the U.S. Food and Drug Administration (FDA) in 1997 and has consistently been shown to significantly improve progression-free and overall survival [33–36]. In the absence of a NHL-specific questionnaire, previous studies investigating the impact of (R-)CHOP or (R-)CVP on QoL of follicular lymphoma or diffuse large B-cell lymphoma have often used generic and non-validated questionnaires and added extra items to cover expected symptoms, like tingling in hands/feet [37, 38]. More recent randomized phase III trials have greatly contributed to a better understanding of the impact of therapy in rituximab-refractory NHL patients [39].

With regard to HL, a recent systematic review [40] has shown a considerable number of studies with QoL as endpoint published over the last few years, with some half of the 65 studies identified, published after 2005. However, the majority of studies used non-HL disease-specific questionnaires. Also, there was a lack of studies documenting QoL outcomes of patients during active treatment as the majority were on patients off treatment.

Furthermore, in recent years, many novel targeted therapies for CLL have been introduced, majorly transforming CLL treatment, although watch and wait remains the standard approach for patients not meeting treatment criteria [41]. Only CLL patients with active or symptomatic disease or with advanced Binet or Rai stages require therapy [42]. For physically fit patients, chemoimmunotherapy with fludarabine, cyclophosphamide and rituximab is currently the standard therapy [43]. For unfit patients, treatment with an anti-CD20 antibody plus milder chemotherapy (chlorambucil) may be applied [42]. Several ongoing and planned phase III trials will determine, whether the novel targeted therapies will further improve outcomes for patients with CLL. For example, Ghia and colleagues have recently shown that in patients with relapsed CLL, treatment with rituximab plus idelalisib provided better QoL outcomes and superior symptom control than rituximab plus placebo [44].

It seems likely that a combination of therapies will increasingly be used for long-term disease control in specific subgroups of patients. While novel agents have demonstrated improvements in disease-free and overall survival, they are also associated with unique toxicities (e.g. neutropenia, leukocytopenia) [43] that have not been previously observed with conventional therapies, requiring careful monitoring and management strategies [45]. In the absence of disease-specific QoL measures, previous landmark trials like the CLL8 evaluated QoL using a generic cancer HRQOL measure such as the EORTC QLQ-C30 [46]. This tool may have possibly limited the full appraisal of treatment outcome differences between arms. Although EORTC QoL group members had previously developed the CLL-16 questionnaire, important developments in CLL treatment urged us to develop a new, up-to-date module that includes symptoms associated with new therapies. With the development of the EORTC-CLL17, we now have a more precise, reliable and responsive measure for the evaluation of the impact of CLL and therapies to HRQOL changes.

As new lymphoma and CLL therapeutics are rapidly evolving, each class of drugs with its own toxicity profile, our newly developed questionnaires may need regular updates to ensure adequate assessment of side effects of treatment. The development of four different modules for the four patient groups increases flexibility when it comes to updating the modules in the (near) future. But, as the process of module updating may still be quite time consuming, the EORTC Quality of Life Group has recently adopted a more flexible approach in the inclusion of items to assess patient-reported outcomes after cancer and its treatment [47]. This approach employs a combination of the EORTC QLQ-C30 questionnaire, a condition-specific module, such as in this paper described and if needed, additional items from the EORTC item library. The EORTC maintains an *Item Library* of 600 items (http://groups.eortc.be/qol/item-bank) in many languages derived from its internationally validated modules, which is available to all interested researchers. Thus, if certain side effects, associated with new drugs, are not covered in the existing lymphoma modules, researchers are encouraged to use the item library to add items to their questionnaire. This flexible approach of combining standardized patient-reported questionnaires with validated items from the EORTC item libraries ensures up-to-date assessment of not only the specific side effects of novel therapies, but also their impact on the common functional health problems reported by patients.

In conclusion, four new EORTC questionnaires for patients with lymphoproliferative disorders have been cross-culturally developed according to highest quality international standards. The proposed scale structure will be tested more rigorously and further validated in the forthcoming international field test to confirm psychometric properties.
